# Novel *cis* compound heterozygous variants in *MYO6* causes early onset of non-syndromic hearing loss in a Chinese family

**DOI:** 10.3389/fgene.2023.1275633

**Published:** 2024-01-11

**Authors:** Haiting Ji, Lichun Zhang, Hafiz Muhammad Jafar Hussain, Ayesha Aftab, Huiqian Yu, Min Xiao

**Affiliations:** ^1^ Department of Otorhinolaryngology, Affiliated Eye and ENT Hospital of Fudan University, Shanghai, China; ^2^ ENT Institute and Otorhinolaryngology, Department of Eye and ENT Hospital, State Key Laboratory of Medical Neurobiology and MOE Frontiers Center for Brain Science, Fudan University, Shanghai, China; ^3^ NHC Key Laboratory of Hearing Medicine, Fudan University, Shanghai, China; ^4^ Department of Otorhinolaryngology, Head and Neck Surgery, Otto Körner, Rostock University Medical Center, Rostock, Germany; ^5^ Molecular and Human Genetics, Baylor College of Medicine, Houston, TX, United States; ^6^ Department of Biological Sciences, International Islamic University, Islamabad, Pakistan; ^7^ Shanghai Ji Ai Genetics and IVF Institute, The Obstetrics and Gynecology Hospital of Fudan University, Shanghai, China

**Keywords:** non-syndromic hearing loss, targeted gene panel sequencing, Sanger sequencing, *MYO6*, *cis* pattern

## Abstract

**Background:** Mutations in the *MYO6* gene have been associated with both autosomal dominant non-syndromic hearing loss (ADNSHL) and autosomal recessive non-syndromic hearing loss (ARNSHL), with a cumulative identification of 125 pathogenic variants. To investigate the underlying genetic factor within a Chinese family affected with heriditary hearing loss, prompted the utilization of high-throughput sequencing.

**Method:** A detailed clinical investigation was performed. Genetic testing was performed by using target panel sequencing, and Sanger sequencing. Targeted sequencing identified the variants and Sanger sequencing was employed to validate segregation of the identified variants within family. Additionally, bioinformatics analysis was performed to strengthen our findings.

**Results:** Clinical investigation revealed the family members were affected by progressive and sensorineural hearing loss with an onset around 8–10 years old. Furthermore, genetic testing identified novel *MYO6* variants, c.[2377T>G; 2382G>T] p.[Trp793Gly; Lys794Asn], positioned in a *cis* pattern, as plausible pathogenic contributors to early-onset hearing loss characterized by a severe and progressive course. Moreover, bioinformatics analysis showd disruptin in hydrogen bonding of mutant amino acids with interactive amino acids.

**Conclusion:** Our research uncovered a relationship between mutations in the MYO6 gene and non-syndromic hearing loss. We identified two variants, c.[2377T>G; 2382G>T] p.[Trp793Gly; Lys794Asn] in *MYO6* as strong candidates responsible for the observed progressive hereditary hearing loss. This study not only adds to our knowledge about hearing problems related to MYO6 but also reveals the presence of monogenic compound heterozygosity. Our study will provide a new sight for genetic diagnosis in such patients and their management for future use.

## Introduction

Hearing loss represents a globally pervasive sensory impairment, exerting profound consequences on social interactions and daily life, and ranking as the third leading contributor to disability. The causes of hearing loss can be intricsic or extrinsic. On a broader view, hearing loss can be classified into acquired and congenital hearing loss. Acquired hearing loss can happen at any point in life and might be due to sickness, a medical condition, or an injury (Lieu et al., 2020). Congenital hearing loss stands as the most prevalent birth defect in the U.S., with an occurrence ranging from 2 to 3 cases of clinically deaf infants for every 1,000 births (CDC Prevention 1999–2007). The severity of congenital hearing loss varies, including mild (26–40 dB), moderate (41–55 dB), moderate-severe (56–71 dB), severe (71–90 dB), and profound (>91 dB). About 50%–60% cases of hearing loss can be due to genetic factors (https://www.cdc.gov/ncbddd/hearingloss/genetics.html).

Hearing loss can be syndromic and non-syndromic. Syndromic hearing loss occurs when a person has hearing loss along with other medical anomalies. Among children with genetic abnormalities leading to hearing loss, about 20% exhibit additional findings alongside their hearing impairment (Gürtler and Lalwani, 2002). On the other hand, non-syndromic hearing loss appears as the only issue without affecting other organ systems (Aldè et al., 2023). The causes of both syndromic and non-syndromic hearing loss include genetic mutations and exposure to substances that can harm development (Cunningham and Tucci, 2017; Aldè et al., 2023).

The prevalence and burden of hearing loss, both globally and in China, underscore the need for comprehensive understanding and effective interventions. More than 5% of the global population, equivalent to 430 million individuals (comprising 432 million adults and 34 million children), are facing hearing loss. Projections suggest that by 2050, the number of people with disabling hearing loss will surpass 700 million, affecting approximately 1 in every 10 individuals worldwide (https://www.who.int/news-room/fact-sheets/detail/deafness-and-hearing-loss). In China, hearing loss is a prevalent health issue, with a considerable impact on public health. A national survey conducted by the Chinese Center for Disease Control and Prevention reported that around 90 million people in China experience some form of hearing impairment (Zhou et al., 2020; Ye et al., 2023).

Sensorineural hearing loss (SNHL) represents a globally pervasive sensory impairment, exerting profound consequences on daily activities. The most common etiological factors of SNHL are environmental and genetic alone or a combination of both (Tian et al., 2018). Genetic mutations are currently considered the leading cause of SNHL both in developed and developing counties, resulting in hereditary hearing loss (HHL) (Tian et al., 2018). Within the realm of non-syndromic cases, hereditary factors account for a substantial proportion, with 80% attributed to autosomal recessive inheritance, 19% to autosomal dominant patterns, and a minor fraction (<1%) linked to mitochondrial, miRNA, and X-linked origins (Shearer et al., 1993; Hone and Smith, 2001). Notably, an up-to-date repository reveals associations of 51 genes with autosomal dominant non-syndromic hearing loss (ADNSHL), primarily implicated in post-lingual auditory impairment, alongside 76 genes implicated in autosomal recessive non-syndromic hearing loss (ARNSHL) (https://hereditaryhearingloss.org). The prevelance of heariditery syndromic and non-syndromic hearing loss in europe is 30% and 70% respectively (Del Castillo et al., 2022). The most known genes for syndromic and non-syndromic hearing loss *GJB2*, *MYO7A*, *USH2A*, *COL4A6*, and *MYO6* etc.

Central to this intricate soundscape of genetic influence, the *MYO6* gene encodes the MYO6 protein, domiciled on the long arm of chromosome 6 (6q14.1) and characterized by a span of 35 exons. MYO6 is a central player in the mechanotransduction processes of the inner ear, facilitating the conversion of mechanical stimuli, like sound vibrations, into electrical signals. It is indispensable for preserving the structural integrity and organization of stereocilia bundles, the hair-like projections critical for detecting mechanical stimuli (Mohiddin et al., 2004; Alkowari et al., 2022). Normal functional MYO6, expresses in the inner hair cell in the cochlea, is a prerequisite for the structural integrity and proper functioning of inner ear hair cells, including endocytosis, ion channel regulation, anchoring of stereocilia and vesicle movement (Self et al., 1999; Hertzano et al., 2008; Oonk et al., 2013) collectively safeguarding the integrity of stereocilia form and function. At the core of MYO6’s functionality lies its motor domain, responsible for ATP hydrolysis and actin binding, crucial for its role as a molecular motor, MYO6’s tail region contains distinctive domains, including the unique insert (insert I), setting it apart from other myosin isoforms (Oonk et al., 2013). Perturbations within the MYO6 domain have been unequivocally implicated in both autosomal recessive non-syndromic hearing loss (DFNB37) and autosomal dominant non-syndromic hearing loss (DFNB22) (Melchionda et al., 2001; Ahmed et al., 2003). The impact of *MYO6* mutations spans diverse populations and ethnic cohorts. Melchionda et al. (2001) discerned a missense mutation in MYO6 that underscored ADNSHL within an Italian family with onset age ranging 8–10 years (Melchionda et al., 2001). A study by Mohiddin et al. (2004) illuminated a heterozygous missense mutation in *MYO6*, precipitating autosomal dominant sensorineural deafness intertwined with familial hypertrophic cardiomyopathy with onset age of diseased symptoms between 5 and 54 years (Mohiddin et al., 2004). Furthermore, Sanggaard et al. (2008) reported a heterozygous nonsense mutation within the *MYO6* gene, presenting ADNSHL within a Danish family without describing onset age (Sanggaard et al., 2008). Paralleling these findings, Hilgert et al. (2008) elucidated a splice site variant in *MYO6*, charting its path to ADNSHL within a Belgian family and onset age was <50 years (Hilgert et al., 2008). Similarly, Tian et al. (2018) reported a missense mutation in *MYO6*, discerning its role in ADNSHL within a Chinese lineage and diseased onset age range was 14–81 years (Tian et al., 2018). Besides this, Oka et al. (2020) reported 27 possibly disease-causing *MYO6* candidate variants, including nonsense, frameshift, splicing, and non-frameshift deletion, in 32 Japanese families having ADNSHL and the average age of onset age was 40 years old (Oka et al., 2020). Ahmed et al. (2003) identified 3 mutations in *MYO6* gene with onset age of 9–21 years including a frameshift mutation, a nonsense mutation, and a missense mutation in 3 Pakistani families segregating with ARNSHL (Ahmed et al., 2003). Altogether, these studies showed a wide range of diseased onset age both in ADNSHL and ARNSHL across the globe.

Besides this, single nucleotide polymorphisms (SNPs) were alos associated with the hearing loss and other diseases (Antonino et al., 2022; Li et al., 2022; Tan et al., 2022). Meta analysis and screening studies for such genetic markes have shown a strong association of SNPs with hearing loss suggesting their important role in normal functioning and development.

Till now, there are 125 variants have been detected, which are thought to be pathogenic and associated with deafness or other phenotypes (https://www.hgmd.cf.ac.uk/ac/gene.php?gene=MYO6, accessed on November 2023) and this number is increasing continuously by the use of advanced sequencing technologies.

In this study, we report novel compound heterozygous *cis* variants in *MYO6* in a Chinese family, which presents ADNSHL, by the use of next-generation sequencing (NGS) analysis and Sanger sequencing.

## Materials and methods

### Subjects

The study cohort consisted of a proband, a 25-year-old female who sought consultation at the Shanghai Ji Ai Genetics and IVF Institute, Obstetrics and Gynecology Hospital of Fudan University, due to hearing loss and pregnancy-related concerns. In addition, a family having hearing loss in three generations was recruited to undergo comprehensive clinical assessment and genetic analysis. ENT examinations were conducted at the Department of Otorhinolaryngology, affiliated with the Eye and ENT Hospital of Fudan University. Pure tone audiometry (PTA) covering frequencies up to 8 kHz and tympanometry were performed for each participant. Both air (0.25, 0.5, 1, 2, 4, 6, and 8 kHz) and bone (0.25, 0.5, 1, 2, 4, 6, and 8 kHz) conduction thresholds were determined to exclude conductive hearing loss. The severity of hearing loss was categorized into five distinct groups: normal hearing (PTA <20 dB hearing level (HL)), mild (20–40 dBHL), moderate (41–70 dBHL), severe (71–90 dBHL), and profound (>91 dBHL) (Mazzoli et al., 2003). Additionally, temporal bone computer tomography (CT) scans were conducted to assess potential middle and/or inner ear malformations. Written informed consent was obtained from all participants, and ethical approval was granted by the Ethics Committee of the Obstetrics and Gynecology Hospital of Fudan University (Approval Code: JIAI E2022-06). The age of onset and the progressive nature of hearing loss were meticulously documented.

### Genetic analysis

Genomic DNA (gDNA) was isolated according to a previously established protocol (Ji et al., 2014). Briefly, peripheral blood samples (3–5 mL) were collected from all participants, and gDNA extraction was carried out using the QIAamp DNA Blood Maxi Kit (Qiagen, Valencia, CA). The quality of gDNA was assessed by measuring the optical density ratio (260/280 ratio) and visualized through gel electrophoresis imaging. DNA library construction commenced with the fragmentation of gDNA (5 μg) into approximately 300 base pairs using the Covaris E210 DNA shearing instrument (Covaris Inc., Woburn, MA). Subsequently, the NEBNext™ DNA Sample Prep Master Mix set (E6040, NEB Biolab, Ipswich, MA) was employed for library preparation. Post-library preparation, gDNA libraries underwent purification and size selection using the AMPure DNA Purification kit (Beckman Agencourt, Danvers, MA). The resulting ligation products (20 ng) were subjected to 14 PCR cycles with Illumina PCR primers InPE1.0, followed by purification using QIAquick MinElute columns. Elution was performed into 50 μL of hybridization buffer (HB, Roche NimbleGen, Madison, WI). Targeted panel sequencing, encompassing 415 genes associated with deafness, was carried out, with the enrichment of target genes evaluated using quantitative PCR (qPCR). Variant classification adhered to the American College of Medical Genetics and Genomics (ACMG) criteria, and identified variants were further validated using Sanger sequencing.

### Sanger sequencing

The validation of candidate pathogenic mutations was performed using Sanger sequencing in both affected patients and their unaffected family members. Sanger sequencing for MYO6 gene mutations was outsourced to a commercial provider (Beckman Coulter Genomics, Danvers, MA). Exon 23 of MYO6 was amplified using the following primers: 5′- CTG​CCA​AGG​CCT​ATG​TAA​TTG​A-3′ and 5′-GGG​ACC​CAG​TGT​GAA​CAA​GTC​T-3’. The interpretation of sequence variants and the assessment of their pathogenicity were guided by the guidelines provided by the American College of Medical Genetics and Genomics (ACMG), the Association for Molecular Pathology (AMP), Sequence Variant Interpretation Working Group (SVI) in ClinGen, and the Association for Clinical Genomic Science (ACGS).

### Bioinformatics analysis

To elucidate the potential impact of the identified variants on the structural and functional aspects of the MYO6 protein, we conducted a comprehensive conservation analysis. The 3D model of MYO6, obtained from the AlphaFold DB (AF-Q9H3D4-F1), served as the foundational template for this analysis (https://alphafold.ebi.ac.uk/entry/Q12951). Leveraging this advanced computational tool enabled us to delve into the intricate spatial arrangement and interplay of amino acid residues within the MYO6 protein.

Moreover, the anticipated 3D structure of the MYO6 protein model was meticulously visualized using PyMOL software (Version 2.5; Schrödinger, LLC). This visualization process facilitated the exploration of potential consequences stemming from the novel compound heterozygous variants at a structural level. Through a close examination of the conformational changes introduced by these variants, our aim was to unravel potential disruptions within critical functional domains and interaction interfaces of the protein. The combined conservation analysis and 3D modeling provided a platform for a deeper understanding of the molecular implications of the identified variants.

## Results

### Clinical description

**FIGURE 1:**
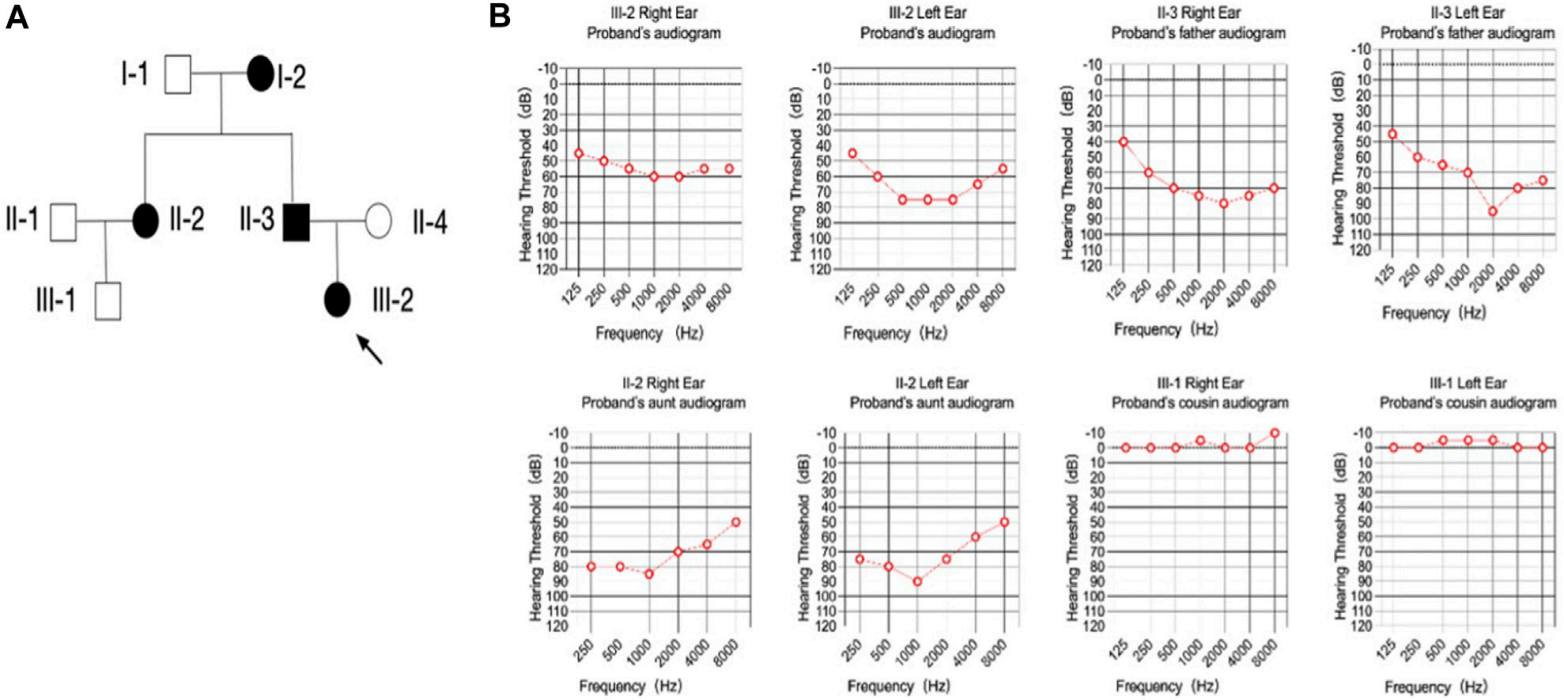
Pedigree and audiometry of the affected family. **(A)** Pedigree of a family affected with non-syndromic hearing loss. The family members are presented as: circle (females); rectangle (males); filled symbols (affected individuals); open symbols (normal individuals); arrow (proband). **(B)** Pure tone of affected individuals. The results are bilateral symmetry and there is no significant difference were found in hearing threshold between the right and left ear. The threshold of the middle frequencies was most affected comparing with the high and low ones, which made the audiogram shaped like a cookie-bite or a letter of ‘U’.

A multigenerational family encompassing four individuals affected by postlingual dominant non-syndromic hearing loss (ADNSHL) was recruited, as depicted in Figure 1A. Audiograms were obtained from all participants to facilitate clinical evaluation. There are a total of 8 family members. Affected individuals (I-2, II-2, II-3, III-2) exhibited sensorineural, bilateral, and progressively worsening hearing impairment, with a notable emphasis on middle frequencies over high and low frequencies (Figure 1B). Remarkably, hearing loss onset for all four individuals occurred around 8–10 years of age, leading to a noticeable decline in quality of life by age 20 and near-complete subjective deafness by age 30 (Figure 1B). Unfortunately, no prior hearing assessments has been conducted before their consultation. Comprehensive physical examinations revealed normal otorhinolaryngology status and an absence of significant neurological disorders. Consequently, a combination of familial history, clinical assessment, and physical examination established the presence of dominat non-syndromic hearing loss among the affected family members.

### Variant identification

**FIGURE 2:**
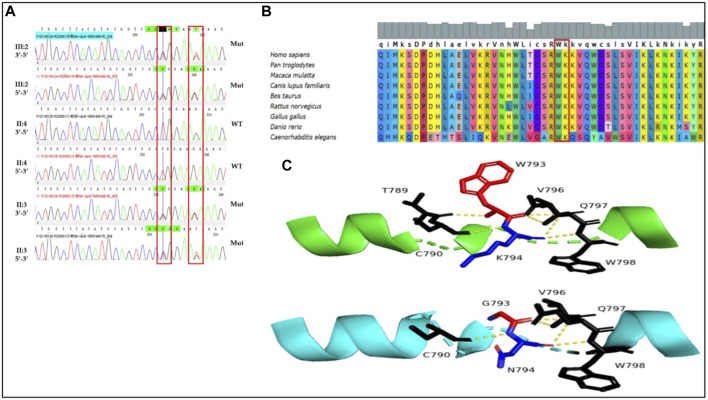
Sanger sequencing and *in silico* analysis of the identified variants. **(A)** Sanger sequencing confirmed the identified variants of *MYO6* (c.2377T>G and c.2382G>T) are in *cis* pattern. Furthermore, the identified variants were not detected in healthy mother (II:4). **(B)** Multiple sequencing alignment showed the wild type amino acids at mutant points were highly conserved from lower to higher species. **(C)** Predicted three-dimensional structure of MYO6. Blue atomic form shows the location of the sites concerned. The variant position at residues 793 and 794 are represented by a ball model. One of the hydrophobic interactions with amino acids seen in the wild-type protein are lost when the amino acid changes in the frame. The top image represents the wild-type MYO6 protein, while the bottom image displays the spatial conformation of the mutated. Residue Trp793 forms tryptophan hydrogen bonds with cysteine and threonine at distances of 3.0 Å and 2.9 Å, respectively. When Trp793 was replaced with glycine, the hydrogen bonds of tryptophan interacting with threonine binding calmodulin region.

In pursuit of unraveling the genetic basis of the condition within this recruited family, we employed targeted panel sequencing focused on 415 genes associated with deafness in proband (III:2). This analysis revealed the identification of four potential mutation loci (Table 1). Intriguingly, mutations within *ADGRV*1 and *TECTA* were excluded as these were observed in some unaffected individuals from databases. So, novel compound heterozygous missense mutations within *MYO6* (c.2377T>G and c.2382G>T) emerged as compelling candidate causative factors. Importantly, these variants were absent from public databases (gnomAD, HGMD, ClinVar and Ensembl). Subsequent segregation validation through Sanger sequencing in available family members (II:3, II:4 and III:2) confirmed inheritance of these variants from affected father (II:3) to daughter (III:2) (Figure 2A). Altogether, all the evidences confrimed, the identified variants may be the causative of disease in patients of the recruited family.

**TABLE 1:** Detected mutations in the affected family.

Variant genes	Coordinate (hg19)	cDNA change	Protein change	Sub-region	Zygosity	Chromosome	Variation type
*MYO6*	NM_004999	c.2377T>G	p.Trp793Gly	EX23/CDS22	Het	Chr6:76591496	Damaging
*MYO6*	NM_004999	c.2382G>T	p.Lys794Asn	EX23/CDS22	Het	Chr6:76591501	Tolerated
*ADGVRV1*	NM_032119	c.199A>G	p.Ile67Val	EX2/CDS2	Het	Chr5:89910828	VUS
*TECT4*	NM_005422	c.5908G>A	p.Ala1970Thr	EX19/CDS19	Het	Chr11:121039543	VUS

### *In-silico* analysis

The conservation analysis of amino acids revealed that at mutant sites the wild type amino acids are highly conserved across a diverse range of species (Figure 2B). Impressively, these identified mutations were strategically positioned within a conserved domain that plays a pivotal role in calmodulin binding, as well as within a helical region of MYO6 (Figure 2C). Leveraging the AlphaFold DB (AF-Q9H3D4-F1) structure model, we found the ramifications of the p.Trp793Gly and p.Lys794Asn variants. As illustrated in Figure 2C, residue Trp793 formed hydrogen bonds with cysteine and threonine at distances of 3.0 Å and 2.9 Å, respectively. Intriguingly, the substitution of Trp793 with glycine disrupted the hydrogen bonds formed with threonine, hinting at a plausible link to the pathogenicity observed in the patients.

## Discussion

In this study, we diagnosed two patients the proband (III-2) and her father (II-3) from a Chinese family with non-syndromic hearing loss. To explore potential genetic causes, we conducted targeted gene panel sequencing. Our analysis revealed two previously unreported heterozygous variants in *MYO6*, specifically c.2377T>G and c.2382G>T. Moreover, Sanger sequencing confirmed that these identified variants exhibited a *cis* pattern of inheritance, transmitted from father to daughter. Significantly, both variants were absent in mother, confirming *cis* inheritance and also absent in public databases.

Our study reveals novel compound heterozygous mutations involving two distinct nucleotides within the *MYO6* gene: c.2377T>G and c.2382G>T, resulting in amino acid substitutions p.Trp793Gly and p.Lys794Asn, respectively. In initial assessment, we propose that the NM_004999.3 (*MYO6*): c.2377T>G (p.Trp793Gly) variant serves as the primary causative factor for deafness, while the second variant, NM_004999.3 (*MYO6*): c.2382G>T (p.Lys794Asn), exhibits weaker pathogenicity. Hearing loss typically a monogenic disorder. According to the ACMG classification criteria, both variants can be classified as uncertain significance.

We can conclude that NM_004999.3 (*MYO6*): c.2377T>G (p.Trp793Gly) is the more likely causative factor, given its higher prediction score by REVEL (0.8719) than NM_004999.3 (*MYO6*): c.2377T>G (p.Trp793Gly) (REVEL score: 0.444).

The mutation c.2377T>G in the *MYO6* gene (NM004999), leading to the amino acid change p.Trp793Gly, is a missense mutation. Notably, this mutation has not been identified previously. Conversely, the second point mutation, c.2382G>T in *MYO6* (NM004999), resulting in the amino acid change p.Lys794Asn, is also a missense mutation. Importantly, this is also absent in databases.

Hence, it is plausible that the first mutation, c.2377T>G in *MYO6* (NM004999), and the corresponding p.Trp793Gly substitution, may be the primary causative factor for the observed deafness, given its rarity and the strong predictions of pathogenicity.

Prior investigations have documented that most MYO6-associated hearing loss presents as sensorineural, bilateral, and progressive. While the hearing threshold typically remains stable until around age 40, a rapid deterioration occurs thereafter, leading to severe hearing loss by age 60. When the analysis was restricted to patients over 40 years of age, the deterioration speed increased to 1.07 dB per year (Miyagawa et al., 2015; Oka et al., 2020). Notably, in our current study, all two cases associated with *MYO6* mutations displayed an earlier onset of hearing loss and a more aggressive progression. It is essential to exercise caution when interpreting these findings, as the limited sample size within this family restricts definitive conclusions. Furthermore, earlier research has suggested that MYO6-related hearing impairment initially impacts high frequencies before progressing to middle and low frequencies. Interestingly, our study’s affected participants, except for the grandmother (I-2) who suffered from deafness, exhibited distinctive characteristics, primarily manifesting as middle frequencies impairment. We speculate that this phenomenon could arise from two potential factors: the presence of the identified *MYO6* variants and the influence of the affected protein domain.

Encoding the myosin VI protein, consisting of 1,285 amino acids, MYO6 plays a pivotal role in maintaining structural integrity, facilitating development, and ensuring proper functioning of inner ear hair cells. Additionally, MYO6 contributes significantly to cargo transport towards the minus end of actin filaments (Wells et al., 1999; Sweeney and Houdusse, 2007). Furthermore, MYO6 localizes to the cuticular plate of hair cells, providing protection against constraining mechanical forces (Avraham et al., 1995; Hasson et al., 1997; Self et al., 1999). This protein mainly comprises a motor domain, a neck domain, and a globular tail that interacts with other proteins (Sweeney and Houdusse, 2007). Topsakal et al. (2010) proposed a genotype-phenotype correlation, suggesting that dominant versus recessive mutations in MYO6 exhibit distinct effects (Topsakal et al., 2010). When the motor domain is impaired and its function compromised, severe early-onset hearing loss occurs. In contrast, missense mutations that do not affect the motor domain lead to milder hearing impairment with a later onset (Topsakal et al., 2010). This trend has been corroborated by Oonk et al. (2013), who observed milder hearing impairment resulting from missense mutations that do not impact known functional protein domains (Oonk et al., 2013). Studies in mice models with *MYO6* mutations have shown that inner hair cell stereocilia appear normal at birth but begin to fuse during the aging process (Seki et al., 2017). This dynamic could elucidate the normal hearing observed during early ages, followed by a deterioration process during aging. Notably, distinct mutation types result in varying phenotypic severities, specifically in terms of age of onset, progression, and severity (Melchionda et al., 2001; Mohiddin et al., 2004; Sanggaard et al., 2008; Topsakal et al., 2010). In our study, the *MYO6* mutations specifically affect the calmodulin binding domain, preserving the motor domain yet leading to early onset and disrupted protein function, ultimately resulting in hearing loss. Consequently, the compound heterozygous variants exhibited faster progression and greater impact compared to previously reported homozygous mutations. Therefore, we posit that the dual mutation points in MYO6 may exacerbate the hearing loss phenotype. Furthermore, digenic compound mutations of *MYO6* have been reported with *ILDR1* in an Iranian family previously (Seki et al., 2017). As such, we report monogenic compound heterozygous mutations of MYO6.

## Conclusion

In conclusion, our study presents a distinctive manifestation of autosomal dominant non-syndromic hearing loss (ADNSHL) in a Chinese family, underscored by the identification of novel compound heterozygous variants within the *MYO6* gene. These variants, c.[2377T>G; 2382G>T] p.[Trp793Gly; Lys794Asn], were ascertained through a combination of targeted gene panel sequencing, Sanger sequencing, and *in silico* analysis. Our findings substantiate the pivotal role of *MYO6* in the etiology of early-onset hearing loss, unveiling a facet of monogenic compound heterozygosity. By characterizing the clinical course and genetic courses, and deciphering the structural implications, our study not only enriches the understanding of MYO6-related auditory pathologies but also provides the insight into the realm of monogenic compound heterozygosity.

## Data Availability

The datasets for this article are not publicly available due to concerns regarding participant/patient anonymity. Requests to access the datasets should be directed to the corresponding authors.
